# Chronic Pain Management During a Pandemic: Evidence-Based Review

**DOI:** 10.5152/TJAR.2022.1328

**Published:** 2022-06-01

**Authors:** Niyati Arora, Ajit Kumar, Ajay Kumar, Ravi Shankar Sharma

**Affiliations:** 1 Department of Anaesthesiology, All India Institute of Medical Sciences, Rishikesh, Uttrakhand

**Keywords:** Chronic pain, coronavirus, COVID-19, drugs usage in COVID-19, guidelines, pain management, recommendation

## Abstract

Chronic pain is the leading cause of morbidity in the world and is strongly associated with physical and psychological disabilities. In this pandemic, most of the pain care centers are forced to shut their doors leaving patients in dismay and adding to their misery. A systematic review was performed following the recommendations of the Preferred Reporting Items for Systematic Reviews and Meta-Analyses statement. All research articles from March 2020 to September 15, 2020, available on PubMed, Google scholar, and EmBase were included in this study. The keywords used for data search were “chronic pain,” “coronavirus,” “pain management,” “COVID-19,” “drugs usage in covid-19,” “recommendation,” and “guidelines”. This review summarizes findings from the current literature available worldwide from different databases regarding guidelines to practice during chronic pain in coronavirus disease (COVID) crisis. This article acts as a specimen on how to handle future pandemics. We concluded that chronic pain management is a fundamental right and telemedicine is the silver lining that can be used for primary, follow-up consultation and to address mental health issues in chronic pain patients. Outpatient department visits should be scheduled using “forward triage.” Pain Interventions should be carried out if urgent or semi-urgent with necessary precautions. Reopening of elective procedures with COVID testing can be planned, considering pain interventions to be usually percutaneous, of short duration, and involving office procedures with minimal aerosol generation. Drugs contributing to immune suppression, for example, strong opioids and steroids, should be avoided. Regenerative therapy can be used instead during pain interventions. Physicians are expected to follow the recommended government guidelines before prescribing any drugs.

**Main Points**

- With so many guidelines and recommendations, a systematic review like this one that summarizes the most noteworthy instructions for pain physicians to practice while chronic pain management in coronavirus disease (COVID) crisis was vital.
- Drugs like steroids, strong opioids (which alter immune response), and non-steroidal anti-inflammatory drugs (which can mask COVID symptoms) should be used cautiously.
- The drug-dosage adjustments and alternate for immune-compromising treatments are discussed.
- Guidelines to “re-open” outpatient services and to perform interventional procedures are enumerated.

## Introduction

Chronic pain patients have the largest global morbidity. The actual burden of chronic pain is diluted due to under-reporting or patients not visiting the medical facility until dismally needed. An Indian study by Saxena et al.^1^ shows a huge chronic pain burden in India by the prevalence rate of 19.3%, which translates into 180-200 million adults having chronic pain. All systematic reviews on chronic pain have concluded that there is an association between pain, mental illness, suicide incidence, and decreased life expectancy.

Coronavirus disease 2019 (COVID-19) has taken the world by storm and was declared as a pandemic by the World Health Organization (WHO) on January 30, 2020. Numerous preliminary research articles have been published about the rapidly spreading coronavirus and its impact on global health. It is juvenile to think of COVID-19 as the last pandemic. The research done in this time can serve as an example to handle future pandemics. We conducted a systematic review to summarize and critically analyze all published scientific articles regarding chronic pain management in this COVID-19 era.

In this systematic review, we aimed to summarize how severe acute respiratory syndrome coronavirus 2 (SARS-CoV-2) impacted chronic pain patients (CPP) and pain physicians. A consensus is drawn on how chronic pain management is being practiced worldwide and what preventive measures health care workers (HCWs) can take while providing pain services in this pandemic.

To understand and discuss:

- role of telemedicine,
- usage of drugs and their doses during COVID-19 pandemic,
- precautions to be taken during outpatient department (OPD) visit and during pain interventions.

## Methods

**Figure 1.**
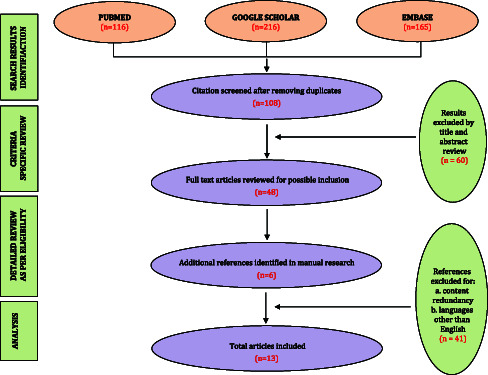
Literature search algorithm.

Four researchers independently searched through the relevant articles published from March 2020 to September 15, 2020. All the selected abstracts were reviewed and analyzed. The literature for this review was identified by searching the following online databases: PubMed, Google scholar, and EmBase. We searched scientific publications using the keywords “chronic pain,” “coronavirus,” “pain management,” “COVID-19,” “guidelines,” and “recommendations” (Figure 1).

Guidelines from Directors of the American Society of Regional Anesthesia (ASRA), American Academy of Pain Medicine, Spine Intervention Society, North American Neuro-modulation Society, and the World Institute of Pain integrated with the American Society of Anasthesiologists (ASA), and American Academy of Physical Medicine and Rehabilitation published in Pain Medicine journal on April 1, 2020, were extensively analyzed. Furthermore, the WHO database of publications on novel coronavirus was screened for potentially relevant publications.^2^ Ministry of Health and Family Welfare, Government of India website, was comprehensively scrutinized and all relevant recommendations were included.^3^ Indian Society for Study of Pain position statement for pain medicine practice during the COVID pandemic published on August 3, 2020, was studied.^4^ This systematic review was performed following the recommendations of the Preferred Reporting Items for Systematic Reviews and Meta-Analyses (PRISMA) statement. As per the PRISMA statement, a systematic review starts from a focused research question, using clearly stated methods to retrieve, assess, and analyze research on a specific topic of interest.

## Results

Around 116 articles were found in the PubMed database, 216 in Google scholar, and 165 in EMBASE. Duplicate entries were removed, after which 108 reports remained. Screening of the reports based on the title and abstract led to the exclusion of 86 studies that did not meet the inclusion criteria. Full texts of 22 studies were retrieved and assessed for eligibility and another 6 articles were included after manual search. This led to the exclusion of 15 studies that did not meet the inclusion criteria for various reasons (Table 1): 6 were duplicate publications, 4 were an erratum of an already included study, 3 turned out to be commentary, and 3 were a letter to the editor. In the end, 13 studies were included in the qualitative synthesis. An overview of the retrieved articles can be found in Supplementary Tables 1 and 2.

### Problems Faced by Chronic Pain Patients

Receiving pain treatment is a fundamental human right, but due to lockdown/travel restrictions patients are not able to reach OPD services for primary or follow-up visits. The healthcare resources are redistributed toward intensive care units and other COVID-19-dedicated sites. Also, because most of the pain physicians worldwide are anaesthesiologists, they are working in COVID areas, thus reducing the availability of pain facilities and pain physicians. Incomplete refill of medicines leads to inadequate pain relief, which then results in physical and mental suffering. Mental health disorders (e.g., depression, anxiety disorders, post-traumatic stress disorder, and substance use disorders) are common in patients with chronic pain.^5^ Wang et al^6^ showed that COVID-19 outbreak and mandatory quarantine have also been associated with negative psychological implications which can exaggerate chronic pain. Thus, mental health considerations are highly relevant in COVID times.

### Problems Faced by Chronic Pain Physicians

In March 2020, the government health authorities of the United States, European Union, United Kingdom, India, and the rest of the world stated that all routine chronic pain services will be shut in view of the pandemic. The majority of pain physicians were quarantined at home or started working as COVID warriors. A survey on pain physician’s burnout during COVID-19 demonstrated that 98% of physician practices were affected by COVID and 91% of physicians felt that it had a significant financial impact.^7^ Quarantined HCWs are more likely to report mental exhaustion, social detachment, feeling of anxiety when treating febrile patients, irritability, sleep disruption, and poor concentration. The probability of alcohol abuse and symptoms of post-traumatic stress disorder also increased for up to 3 years following the episode of quarantine.^8^ In this ongoing pandemic, both patients and HCWs should be exposed to screening and treatment interventions for mental health problems using online and mobile-based platforms.^9^

## Discussion

### Role of Telemedicine

Before the COVID-19 pandemic, telemedicine approaches were being tested but were not prevalent. Suddenly, however, treating patients with non-urgent and long-term conditions in a safe and effective way has become essential. Randomized controlled trials have demonstrated high levels of satisfaction, convenience, and acceptance of e-health services in remotely located patients.^10^ There is an enormous potential for cost reduction and time savings with such services. Telemedicine can be used for “forward triage” in COVID times. Using this, the patient is put in touch with the emergency department to discuss respiratory symptoms and with a pain physician for pain symptoms. This allows patients to be screened efficiently and decide if an in-person visit is needed. It is both patient-centered and conducive to self-quarantine, and it protects patients, clinicians, and the community from exposure.^11,12^

Pain physicians from United Kingdom and Australia emphasized shifting focus to e-health^13,14^ and China concluded that for patients with different types of chronic pain, telemedicine support in addition to necessary in-person visits.^15^ In India, the Board of Governors in supersession of the Medical Council of India in partnership with National Institution for Transforming India Aayog on March 25, 2020, came up with telemedicine guidelines for registered medical practitioners (RMP).^16^ The guidelines state that RMP can practice telemedicine. Modes that can be used include video (telemedicine facility, apps, video on chat platforms, skype/facetime, etc.), audio (phone, voice over internet protocol, apps, etc.), text-based and chat-based applications (specialized telemedicine smartphone apps, websites, other internetbased systems, etc.), general messaging/text/chat platforms (WhatsApp, Google Hangouts, Facebook Messenger, etc.), and asynchronous communication (email/Fax, etc.). A digital trail including summary of patient complaints and supplementary data including images, lab reports, and/or radiological investigations are shared between the doctor and the patient has to be recorded as documentation of proof.

In addition, many international medical services use web-based systems that have been developed and optimized for people with pain, such as the Collaborative Health Outcome Information Registry (CHOIR) system in the United States^17^ or the PAIN OUT system in Europe.^18^ Chronic pain management depends on good clinical history and clinical evaluation. Short Form McGill Pain Questionnaires and Pain Disability Index are examples of electronic pain questionnaires. The validity and dependability of these tests have been proven with significantly more patients reporting electronic versions easier and favored.^19^ Similarly, many questionnaires for the assessment of patients with chronic low back pain have shown good reliability and moderate validity.^20^ The advantage of telemedicine is that red flag signs in chronic pain can be picked up easily during online history taking. Although lack of hands-on physical examination is a limitation with telehealth, a modified virtual examination may allow an initial treatment plan to be started.^21^

The practical issues in telemedicine for CPPs, for example, the absence of apps/video conference programs installed on the patient’s computer/phone, inaccessibility to a high-speed internet connection, an improperly charged mobile phone, laptop, or computer, and lack of apps to record symptoms on a daily basis may lead to failure of the system. Another challenge for telemedicine, especially in Indian government hospitals, includes the inadequate number of functional telemedicine units. All India Institute of Medical Sciences (AIIMS), Rishikesh, Uttrakhand, India, has a dedicated telemedicine OPD that caters to 50-70 CPPs per day in COVID catastrophe.

### Drug Usage in COVID-19

Patients with COVID-19 undergo signiﬁcant immune system changes. The importance of intact immune response to avoid infection and the association between immune system and various drugs has to be kept in mind while prescribing these drugs.

### Opioids

Opioids are the most effective treatment for acute and chronic pain and have been extensively used for both neuropathic and non-neuropathic pain. Opioids act on the autonomic nervous system and hypothalamic–pituitary–ad renal axis and thus inhibit the innate and acquired immune response.^22^ The target should be not to create an opioid withdrawal state due to scarcity of medication and at the same time avoid abuse of opioids. Morphine and fentanyl have been observed as potent immunosuppressants.^23, 24^ Based on available data, buprenorphine appears to be the safest to use in immune-compromised or elderly patients.^25 [Bibr b30-tjar-50-3-159]^

In the United States, health care providers are permitted to issue electronic opioid prescriptions, via telehealth visits, which may reduce the risk of diversion to illicit forms of opioids. Opioid-naïve patients if needed should be started on short-term interim courses. If an opioid requirement is high after 1-2 weeks, an in-person physical examination is recommended.^26^ In patients already receiving opioids, a temporary increase in opioid dose may be provided if desperately needed. But an in-person visit must be scheduled within 2 months to check for opioid tolerance or opioid hyperalgesia. Monitoring of opioid withdrawal in patients is more perplexing during e-health visits. Symptoms like diarrhea, rhinorrhea, abdominal pain, chills and signs like agitation, diaphoresis, piloerection, and possibly even change in pupillary size can be observed remotely during patient interviews but may be difficult to corroborate. However, the physician has to rely on patients or their caregivers to look for classic signs of opioid withdrawal, that is, elevated heart or pulse rate. Non-opioid drugs like clonidine and lofexidine can be prescribed to prevent physical withdrawal symptoms. It is suggested to consider prescribing naloxone in patients with the potential to overdose and counting opioid tablets dispensed over videoconferencing.^27^ Educating patients and relatives about overdose and withdrawal plays a key role in this tricky situation. Patients with COVID-19 who are receiving opioids can be more susceptible to respiratory depression. It is recommended to carefully monitor respiration and sedation in patients on transdermal opioids (fentanyl), because the rate of absorption with high fever can be unpredictable.^28^

In India, MoFHW^16^ states that opioids fall under the prohibited drugs and are to be prescribed via an in-person visit by licensed practitioners only. This is a limitation to telemedicine as many drugs prescribed for CPPs fall in the category of prohibited drugs for e-consultation. AIIMS, New Delhi, India, shifted from prescribing opioid medications from a strict 2-week policy to a 1-month policy and practiced telemedicine to monitor opioid use.^29^ This was done in the best interest of patients as travel restrictions in India were the biggest barrier for patients to reach the hospital. The same policy is being followed at AIIMS, Rishikesh, Uttrakhand, India.

### Steroids

Chronic pain patients consume oral steroids or receive steroid injections for pain syndromes or multiple musculoskeletal diseases. Steroids suppress the immune system and systemic steroids have been associated with infections, including pneumonia. The hypothalamic–pituitary–adrenal axis suppression typically lasts for less than 3 weeks but may last for over 1 month in some individuals. The effects of intra-articular steroids on COVID-19 have not been evaluated; therefore, intra-articular steroids can be used for pain relief when required after assessing the risks and benefits.^30^

The Faculty of Pain Medicine of the Royal College of Anaesthetists’ statement issues caution on the safety of steroids injected during the current COVID-19 pandemic.^31^ In patients with rheumatoid arthritis, oral use of corticosteroid dose of ≥20 mg (0.5 mg kg^-1^) prednisolone (or equivalent) per day for more than 4 weeks, corticosteroid dose of ≥5 mg prednisolone (or equivalent) per day for more than 4 weeks plus at least 1 other immunosuppressive medication (biologic/monoclonal) or small molecule immunosuppressant (e.g., Januse Kinase inhibitors) are extremely vulnerable to COVID-19 infection and needs “shielding.”^32^

Epidural and other steroid injections can be performed as per clinical conditions during the COVID-19 pandemic with the lowest dose possible. Patients should be educated about immunosuppression and the potential risk for infection. For patients who are already at high risk for SARS-CoV-2 infection or are immunosuppressed, epidural non-steroid (e.g., local anesthetics with additives mentioned above) injections may be considered for radicular pain. Considering immunosuppression, oral steroids should not be prescribed as a first-consult drug; but if a patient is already on steroids, then it should be continued. Dose reduction should be considered after evaluating disease conditions.

### Non-Steroidal Anti-inflammatory Drugs (NSAIDS)

Non-steroidal anti-inflammatory drugs remain the most controversial drug in the COVID crisis. Their analgesic effect is due to action on cyclo-oxygenase enzymes and causes peripheral inhibition of prostaglandin synthesis. Scientists believed that drugs such as ibuprofen could exacerbate the COVID-19 disease by upregulating the angiotensin-converting enzyme 2 (ACE2), which serves as an entry receptor for SARS-CoV-2.^33^ On March 18, 2020, the WHO advised patients experiencing COVID-19 symptoms to avoid the use of ibuprofen and other NSAIDS due to the same reasons. However, the effect of NSAIDS on ACE-2 enzyme has not been substantiated by any other reports and multiple regulatory bodies have since refuted this assertion.^34-36^ Acetaminophen is an alternative to NSAIDs. When compared in osteoarthritis patients, it is not only less effective but also less toxic than systemic NSAIDs. Hence, acetaminophen is the most appropriate first-line oral analgesic for long-term in these patients.^37^ Joint guidelines from ASRA/ESRA (European Society of Regional Anaesthesia and Pain Therapy) recommended that all patients who have been prescribed or use NSAIDS on a daily basis can continue using them but to promptly report mild fever or new myalgia.^38^

### Regenerative Medicine

The body has an innate ability in treating its own pathology by repairing damaged tissue using autologous or allogeneic biologics. This ability is reinforced using regenerative medicine. There is variable evidence in favor of mesenchymal stem cells and platelet-rich plasma used in treating discogenic pain, radicular pain, facial joint pain, and sacroiliac joint pain.^39^ It is also proved to be an effective treatment for osteoarthritis of the knee and pelvis by improving the clinical condition and reducing pain. Platelet-rich plasma can be a better alternative to tendinopathy than steroid therapy. Platelet-rich plasma and bone marrow aspirate concentrate are among the orthobiologic therapies used as alternatives to current therapies for muscle, bone, and cartilage injuries.^40^ Further research on the aforementioned regenerative therapies seems necessary during the COVID-19 pandemic to find a good alternative to steroids and opioids.

**Precautions to Be Taken During In-Person Visit to Outpatient Department**

As all the countries have opened up again after months, the piled-up OPD load can be back-breaking. The susceptibility of CPP could be higher as many are elderly with multiple comorbidities and potential immune suppression due to ongoing drug therapies like opioids and steroids. Thus, pain practitioners should rely on telemedicine for preliminary patient interviews then triage into 3 different levels^41^ (Table 2).

On April 7, 2020, Centers of Medicare and Medicaid Services (CMS) recommended a 3-tier approach for triaging all non-essential medical services and procedures by acuity: (1) low acuity or elective (postpone); (2) intermediate acuity or urgent (consider postponement); and (3) high acuity or emergent (do not postpone). 

Official Journal of the American Academy of Pain Medicine points out the following recommendations that are most applicable to interventional pain physicians:

1. Place signs at triage points on appropriate hand hygiene, respiratory hygiene, and cough etiquette.
2. Triage patients with fever and or respiratory symptoms and provide facemasks to symptomatic patients (and also asymptomatic patients if possible).
3. Limit unnecessary patient escorts.
4. Create an area for spatially separating patients ideally at least 6 feet apart in waiting rooms.
5. Patients should be seen in a clean room, with no prior exposure to COVID-19 patients. If patients with COVID-19 or those suspected of having COVID-19 have been in the room, the room needs to be adequately disinfected. These patients should ideally be seen in a separate room.
6. Hand hygiene should be performed with an alcohol-based hand rub for 15 seconds or with soap and water for at least 20 seconds before and between all patient care episodes.
7. Strongly consider the use of surgical, procedural, or cloth face masks on patients during the interaction.
8. In areas with community spread, providers can change into scrubs before seeing patients and out of scrubs before leaving the hospital.
9. Avoid touching one’s face during exposures.
10. Wear gloves during patient care and remove and discard gloves when leaving the care area and immediately perform hand hygiene.
11. Health care providers can wear surgical masks for OPD interaction. N95 masks can be used for “high-risk patients.” Limited reuse of N95 is permissible during times of shortage. In such a scenario, cleanable face shields over the N95 mask should be used to reduce the chance of contamination.
12. Clean and disinfect all surfaces in the patient care environment including tables, beds, chairs, door handles, and equipment between each patient encounter.

In India, quarantine is being lifted in many parts of the country, despite the extensive community spread of coronavirus. The aforementioned precautions can be easily implemented inside the health care setup. Once in community spread, all patients must be considered “high risk.” Considering all patients to be asymptomatic COVID carriers, the number of patients seen per day can be limited. Based on the latest data, the brief contact, such as during a pain medicine consult, confers little risk of transmission from an asymptomatic carrier if precautions are not taken.

### Precautions During Interventions

On March 27, ASRA and ESRA guidelines^42^ stated that only specific urgent, semi-urgent procedures should be performed in designated rooms which should be properly disinfected later. The categorization of pain procedures as elective, urgent, and emergent is in many cases subjective. Though most procedures are characterized as elective, intrathecal pump (ITP) refills and malfunction, and neurostimulator infection and malfunction are considered “urgent.” Guidelines suggest avoiding the insertion of both new intrathecal pumps (except for highly selective cancer patients) and neurostimulators. Pre-encounter briefings, simulation sessions using telemedicine if needed, and the presence of an observer during the removal and discarding of protective gear are highly recommended. The American Society of Pain and Neuroscience COVID-19 Task Force endorses that if the locality or region has sufficient medical resources and personalized protective equipment (PPE) to handle current and/or near-term projected COVID-19 cases, clinicians can proceed with all urgent and urgent elective interventional pain procedures while employing proper social distancing, screening, and testing recommendations.^43^

Semi-urgent procedures are those involved to treat:

1. intractable cancer pain,
2. acute herpes zoster or sub-acute, intractable post-herpetic neuralgia,
3. acute herniated disc and/or worsening lumbar radiculopathy,
4. antractable trigeminal neuralgia,
5. early complex regional pain syndrome,
6. acute cluster headaches and other intractable headache conditions, and
7. other intractable medically resistant pain syndromes (should be reviewed on a case-by-case basis).

On May 18, however, an interim guideline issued by WHO stating “the prior recommendation that all elective procedures be postponed” has been removed. Thus, more in-patient visits and in turn more interventions are likely to happen, and care must be taken to protect both HCW and patients. Preoperative screening followed by testing (if required) at least 48 hours prior to the procedure is suggested. Testing should be done in symptomatic patients, patients with a recent history of contact with COVID-19 patients, patients belonging to “hot spot” area, patients requiring procedure around the airway, and patients requiring general anaesthesia. Interventional pain procedures can be categorized based on 3 factors: percutaneous versus incisional/surgical procedure; short versus prolonged neuraxial entry; and aerosol-generating procedures versus non-aerosol-generating procedures. For the most common procedures performed, patients are positioned prone, and the contact area is limited and sterilely prepped. Most percutaneous interventional pain procedures are performed using a surgical cap, mask, and gloves, without a gown. Examples include ESIs and peripheral nerve blocks. When an individual is performing a percutaneous procedure with prolonged neuraxial access, such as spinal cord stimulation trial or kyphoplasty, it is recommended that a gown be used, in addition to the aforementioned barriers.^44^ N95 masks should be used as recommended by the Centers for Disease Control and Prevention and ASA when procedures with the potential for aerosolization such as intranasal sphenopalatine ganglion blocks and intra-oral injections are performed.^45^ Only urgent or emergent cases should be performed for high-risk patients, as outlined above. These procedures should be conducted in a specially designated room (e.g., negative pressure room). After the procedure, the patient should be monitored in the same room and then transferred to an appropriate isolation area or discharged home to shelter in place. The use of complete PPE with proper donning and doffing is advocated. Equipments like the ultrasound machine and ITP or programmer should be protected from contamination using an appropriate cover. In addition, it is important to ensure that necessary medications (e.g., ITP reﬁll) and equipment are ready and transported in a fully covered plastic bag and handled with sterile gloves in a clean area. Radiofrequency ablation without prior diagnostic blocks and spinal cord stimulation can be allowed without a trial.

## Conclusions

Chronic pain plays a crucial role in an individual’s physical and psychosocial well-being, and access to pain management services is a fundamental human right. In the current scenario of the COVID-19 pandemic, the burden of chronic pain has increased manifold. Precautions at OPD and during interventional procedures are of the utmost importance to avoid healthcare setup from becoming nidus of infection. Washing/sanitizing hands in between checking 2 patients and maintaining standard hygiene cannot be emphasized enough. Mental health care should be given its due significance and should be sorted by both patients and pain physicians. Drug alterations should be done depending on risk–benefit ratio. Telemedicine should be exploited to provide OPD consultation, patient procedural education, and pre/post-procedural consultation.

## Figures and Tables

**Table 1.** Inclusion and Exclusion Criteria

Inclusion Criteria	Exclusion Criteria
Available in the databases on September 15, 2020	Conference abstracts
Published in peer-reviewed journal dissertations in English	Running commentary
Explicitly discuss the management of chronic pain in the COVID-19 crisis	Letter to the editor
COVID-19, coronavirus disease 2019.	

**Supplementary Table 1.** Overview of Retrieved Articles on Chronic Pain in COVID-19

Author	Date of Publishing	Title	Article Type	Most Important Findings
Harsha Shanthanna et al.	March 2020	Recommendation on chronic pain practice during the COVID-19 pandemic	A joint statement by the American Society of Regional Anesthesia and Pain Medicine (ASRA) and European Society of Regional Anaesthesia and Pain Therapy (ESRA)	Chronic pain patients have a high susceptibility to infection due to old age and immune suppression Dose management of opioids and steroids in view of immune suppression Elective, in-person patient visits, or elective procedures to be postponed “Urgent” and “semi-urgent” pain patient procedures to be performed during the COVID-19 pandemic Use telemedicine to evaluate and continue prescriptions Procedural precautions and conduct of procedure for low-risk and high-risk COVID-19 patients
Brook Calton	March 2020	Telemedicine in the time of coronavirus	COVID-19 article	Telemedicine setup, rules, and scope in United States Patient considerations to use telemedicine Clinician considerations to use telemedicine
H. Shanthanna et al.	April 2020	Caring for patients with pain during the COVID-19 pandemic: consensus recommendations from an international expert panel	Original article	Immune suppression with opioids and steroid use Integrating biopsychosocial model in COVID-19 times Planning in-person visits Role of telemedicine Precautions during procedures Urgency to perform intrathecal pump refill or malfunction and neurostimulator removal in case of infection.
COL (ret) Steven P. Cohen et al.	April 2020	Pain management best practices from multispecialty organizations during the COVID-19 pandemic and public health crises	Official Journal of the American Academy of Pain Medicine	Provide a framework for pain practitioners and institutions in alliance with CDC guidelines for health care set-ups Use of 3-layered masks by patients and HCWs at all times Use of N95 for emergent patients and high-risk patients and during procedures that cause aerosolization High-risk patient to be attended only in emergent and urgent condition with consultation and procedure in separate rooms and later sanitation Limited staffing to avoid “unnecessary exposure” Reinforcement of telemedicine Screening of patients before OPD visit Triage to see patients in-person based on acuity, comorbid psychiatric and social considerations, pain level and accompanying functional impairment
Christopher Eccleston	May 2020	Managing patients with chronic pain during the COVID-19 outbreak: considerations for the rapid introduction of remotely supported (eHealth) pain management services	Topical review	Definitions and terminology used in remotely supported pain management, for example, telehealth, telemedicine, e-health, m-health, virtual reality, augmented reality, digital therapeutics (DTx) Distance assessment and treatment using technology Evidence for efficacy and harm of telemedicine and DTx interventions Practical recommendations for the rapid introduction of remotely supported pain management

CDC, Centers for Disease Control and Prevention; HCW, Health care workers.

**Table:** 

**Supplementary Table 2.**Overview of Retrieved Articles on Chronic Pain in COVID-19				
Flaminia COLUZZI	May 2020	Managing chronic pain patients at the time of COVID-19 pandemic	Editorial article	Drug-dose alteration for opioids, steroids, and NSAIDS during COVID-19 Device therapy involving intrathecal pumps and neuromodulatory devices
Babita Ghai et al.	June 2020	Telemedicine for chronic pain management during COVID-19 pandemic	Special article	Scope of telemedicine in India Telemedicine guidelines as published by NITI AAYOG with BOARD OF GOVERNORS in supersession of the Medical Council of India Advantages and limitations of telemedicine Challenges of telemedicine in India
F. Puntillo et al.	July 2020	Impact of COVID-19 pandemic on chronic pain management: looking for the best way to deliver care	Review article	Chronic pain patients features and challenges of pain treatment outside and during the COVID-19 pandemic Recommendations for best practice management of pain patients Use of telemedicine during coronavirus infection pandemic
Timothy Deer, MD	August 2020	Emergence from the COVID-19 pandemic and the care of chronic pain: guidance for the interventionalist	COVID-19 article	American Society of Pain and Neuroscience (ASPN) COVID-19 Task Force to evaluate the policies set forth by federal, state, and local agencies to reduce or eliminate elective procedures for pain patients Sets forth a strategy for the interventional pain centers to reemerge from the current pandemic and to set a course for future events Focuses on screening and testing patients before in-person visits/procedures in the wake of “reopening” Utilization of telemedicine for pre- and post-procedure evaluations
Siddharth Verma et al.	August 2020	Indian society for the study of pain position statement for pain medicine practice during the COVID pandemic	Review article	Discusses burden of pain in COVID-19 Setting up OPD/pain clinic Pain center/clinic reopening checklist for OPD/procedures during COVID-19 pandemic Pain triage: how to differentiate between red flag situations, emergent, urgent, and elective chronic pain conditions based on acuity, comorbid psychiatric (e.g., severe pain-related depression) and social (e.g., single mother of young children with limited resources) considerations Use of telemedicine for triaging Advantages and challenges of telemedicine in Indian setup Resume pain practice with the following goals: avoid deterioration of function; reliance on opioids; or avoidance of unnecessary visits that increase the risk of exposure Interventional pain procedures in low-risk/COVID positive/high risk patients Use of drugs in COVID-19: NSAIDs, STEROIDS, OPIOIDS
Salah N. El-Tallawy et al.	August 2020	Pain management during the COVID-19 pandemic	Review article	Telemedicine as a valuable tool Allows 2-way communication Facilitate follow-up visit Less efficacious for primary visit and physical examination Recommendations for the outpatient pain clinics- patient classified to Level 1, 2, and 3 Recommendations for in-patients to avoid provider to patient and vice-versa spread of infection Multimodal drug therapy would improve outcome with less side-effects NSAIDs, opioids, steroids Transdermal patch discouraged Intrathecal pump refill of opioid is classified as level 3 Interventional pain therapy in COVID-19 Management of pain in patients with COVID-19
**Supplementary Table 2.**Overview of Retrieved Articles on Chronic Pain in COVID-19 (Continued)				
Helen Gharaei, and Sudhir Diwan	August 2020	COVID-19 pandemic: implications on interventional pain practice—a narrative review	Focused review article	Immune suppression increases risk of COVID-19 Immune suppression due to cortisol depends on the type and dose of steroids used Short-acting steroid with the lowest effective dose or use of LA alone without steroids or using another analgesic is more reasonable during COVID-19 time Local anaesthetics used in combination with additive drugs like epinephrine, alpha-2 agonist (clonidine, dexmedetomidine), neostigmine, sodium bicarbonate, adenosine, magnesium, hyaluronidase, etanercept, and tocilizumab is encouraged Dextrose and normal saline both have analgesic properties and should be used as an option Regenerative medicine, for example, mesenchymal stem cells and platelet-rich plasma (PRP) are effective in treating discogenic pain, radicular pain, facial joint pain, and sacroiliac joint pain
Christopher Gharibo et al.	August 2020	Triaging interventional pain procedures during COVID-19 or related elective surgery restrictions: evidence-informed guidance from the ASIPP	Guidelines	Triaging interventional pain procedures during the COVID-19 pandemic The recommendations have been developed using the principles of best evidence synthesis developed by the Cochrane review, incorporating multiple guidelines modified by ASIPP: 1. COVID-ARMS flow chart to mitigate risks of COVID-19 morbidity during interventional pain encounters 2. COVID-ASIPP Risk Mitigation and Stratification (COVID-ARMS) risk stratification of patients presenting for interventional pain procedures based on age, pulmonary, cardiac, renal, hepatic, and immune status. Obesity and diabetes are also considered and patient is classified as low, moderate, or high risk 3. If patient’s residence status is nursing home or assisted living facility or incarceration during the past 30 days, they are considered as high-risk patient. 4. Rating for strength of recommendation into strong moderate and weak 5. ASIPP guidance for triaging pain interventions with examples

NSAIDS, Non steroidal anti-inflammatory drugs; ASIPP, The American Society of Interventional Pain Physicians

**Table 2.** Triage of Patients into 3 Different Levels

Level 1	Patients with mild to moderate pain Clear etiology, pathogenesis, and diagnosis Had relatively well-controlled comorbidities This group of patients can receive pain medication at home, along with telemedicine/eHealth support
Level 2 (e.g., COVID-19-suspected patients)	Level 1 patients who have been exposed to COVID-19-positive individuals Recently traveled to or from an epidemic area, as determined by the World Health Organization (WHO) Have symptoms suggestive of COVID-19, including fever, night sweats, respiratory symptoms, and others Those patients who should self-quarantine at home and should strongly consider getting tested for COVID-19
Level 3	Those with severe pain and/or suspicion of emergency conditions (i.e., spinal fracture, cauda equina syndrome) Those patients who are to receive immediate treatment in the clinic or should be admitted as in patients for further testing and treatment

COVID-19, coronavirus disease 2019.

## References

[1] Saxena AK Jain PN Bhatnagar S . The prevalence of chronic pain among adults in India. Indian J Palliat Care. 2018;24(4):472 477. 10.4103/IJPC.IJPC_141_18) 30410260 PMC6199848

[2] https://www.who.int/emergencies/diseases/novel-coronavirus-2019/situation-reports.(Last Assessed on 14 Sept, 2020)

[3] https://www.mohfw.gov.in/ (Assessed. On. 14th September, 2020).

[4] Verma S Surange P Kothari K et al. Indian society for study of pain position statement for pain medicine practice during the COVID pandemic. Indian J Pain. 2020;34:71 84.

[5] Hooten WM Chronic pain and mental health disorders: shared neural mechanisms, epidemiology, and treatment. InMayo Clinic Proceedings. 2016;91(7):955 970). 10.1016/j.mayocp.2016.04.029 27344405

[6] Wang C Pan R Wan X et al. Immediate psychological responses and associated factors during the initial stage of the 2019 coronavirus disease (COVID-19) epidemic among the general population in China. Int J Environ Res Public Health. 2020;17(5):1729. 10.3390/ijerph17051729) PMC7084952 32155789

[7] Jha SS Shah S Calderon MD Soin A Manchikanti L . The effect of COVID-19 on interventional pain management practices: a physician burnout survey. Pain Phys. 2020;23(4S):S271 S282. 32942787

[8] Wu P Liu X Fang Y et al. Alcohol abuse/dependence symptoms among hospital employees exposed to a SARS outbreak. Alcohol Alcohol. 2008;43(6):706 712. 10.1093/alcalc/agn073) 18790829 PMC2720767

[9] Duan L Zhu G . Psychological interventions for people affected by the COVID-19 epidemic. Lancet Psychiatry. 2020;7(4):300 302. 10.1016/S2215-0366(20)30073-0) 32085840 PMC7128328

[10] O’Brien KM Hodder RK Wiggers J et al. Effectiveness of telephone-based interventions for managing osteoarthritis and spinal pain: a systematic review and meta-analysis. PeerJ. 2018;6:e5846. 10.7717/peerj.5846) PMC6214231 30397549

[11] Zachrison KS Boggs KM Hayden EM Espinola JA Camargo CA Jr . Understanding barriers to telemedicine implementation in rural emergency departments. Ann Emerg Med. 2020;75(3):392 399. 10.1016/j.annemergmed.2019.06.026) 31474481

[12] Hollander JE Carr BG . Virtually perfect? Telemedicine for Covid-19. N Engl J Med. 2020;382(18):1679 1681. 10.1056/NEJMp2003539) 32160451

[13] Calton B Abedini N Fratkin M . Telemedicine in the time of coronavirus. J Pain Symptom Manage. 2020;60(1):e12 e14. 10.1016/j.jpainsymman.2020.03.019) PMC7271287 32240756

[14] Eccleston C Blyth FM Dear BF et al. Managing patients with chronic pain during the COVID-19 outbreak: considerations for the rapid introduction of remotely supported (eHealth) pain management services. Pain. 2020;161(5):889-893. 10.1097/j.pain.0000000000001885) PMC7172975 32251203

[15] Song XJ Xiong DL Wang ZY Yang D Zhou L Li RC . Pain management During the COVID-19 pandemic in China: lessons learned. Pain Med. 2020;21(7):1319 1323. 10.1093/pm/pnaa143) 32321173 PMC7188156

[16] https://www.mohfw.gov.in/pdf/Telemedicine.pdf. Assessed. On. 28 April, 2020.

[17] CHOIR. https://choir.stanford.edu/Assessed. Assessed. On. 8 May, 2020.

[18] PAINOUT. http://pain-out.med.uni-jena.de./Assessed. Assessed. On. 8 May, 2020.

[19] Cook AJ Roberts DA Henderson MD Van Winkle LC Chastain DC Hamill-Ruth RJ . Electronic pain questionnaires: a randomized, crossover comparison with paper questionnaires for chronic pain assessment. Pain. 2004;110(1-2):310 317. 10.1016/j.pain.2004.04.012) 15275781

[20] Azevedo BR Oliveira CB Araujo GMD et al. Is there equivalence between the electronic and paper version of the questionnaires for assessment of patients with chronic low back pain? Spine. 2020;45(6):E329 E335. 10.1097/BRS.0000000000003281) 31593061

[21] Al Hussona M Maher M Chan D et al. The virtual neurologic exam: instructional videos and guidance for the COVID-19 era. Can J Neurol Sci. 2020;47(5):598 603. 10.1017/cjn.2020.96) 32434626 PMC7347716

[22] Mellon RD Bayer BM . Evidence for central opioid receptors in the immunomodulatory effects of morphine: review of potential mechanism (s) of action. J Neuroimmunol. 1998;83(1-2):19 28. 10.1016/s0165-5728(97)00217-8) 9610669

[23] Page GG Immunologic effects of opioids in the presence or absence of pain. J Pain Symptom Manage. 2005;29(5)(suppl):S25 S31. 10.1016/j.jpainsymman.2005.01.006) 15907644

[b30-tjar-50-3-159] Shavit Y Ben-Eliyahu S Zeidel A Beilin B . Effects of fentanyl on natural killer cell activity and on resistance to tumor metastasis in rats. Dose and timing study. Neuroimmunomodulation. 2004;11(4):255 260. 10.1159/000078444) 15249732

[25] Pergolizzi J Böger RH Budd K et al. Opioids and the management of chronic severe pain in the elderly: consensus statement of an International Expert Panel with focus on the six clinically most often used World Health Organization Step III opioids (buprenorphine, fentanyl, hydromorphone, methadone, morphine, oxycodone). Pain Pract. 2008;8(4):287 313. 10.1111/j.1533-2500.2008.00204.x) 18503626

[26] Dowell D Haegerich TM Chou R . CDC guideline for prescribing opioids for chronic pain—United States, 2016. JAMA. 2016;315(15):1624 1645. 10.1001/jama.2016.1464) 26977696 PMC6390846

[27] Shanthanna H Strand NH Provenzano DA et al. Caring for patients with pain during the COVID-19 pandemic: consensus recommendations from an international expert panel. Anaesthesia. 2020;75(7):935 944. 10.1111/anae.15076) 32259288 PMC7262200

[28] Coluzzi F Marinangeli F Pergolizzi J . Managing chronic pain patients at the time of COVID-19 pandemic. Minerva Anestesiol. 2020;86(8):797 799. 10.23736/S0375-9393.20.14666-2) 32400999

[29] Adhikari SD Gupta N Sharma A Deo SVS Bhatnagar S . Caring of cancer patients during COVID-19: a real-life challenge. Indian J Cancer. 2020;57(2):218 220. 10.4103/ijc.IJC_342_20) 32445329

[30] Tan SHS Hong CC Saha S Murphy D Hui JH . Medications in COVID-19 patients: summarizing the current literature from an orthopaedic perspective. Int Orthop. 2020;44(8):1599 1603. 10.1007/s00264-020-04643-5) 32445030 PMC7244258

[31] FPM response to concern related to the safety of steroids injected as part of pain procedures during the current COVID-19 virus pandemic. Available at: https://fpm.ac.uk/sites/fpm/files/documents/2020-03/FPM-COVID-19-Steroid-Statement-2020.pdf.

[32] Price E MacPhie E Kay L et al. Identifying rheumatic disease patients at high risk and requiring shielding during the COVID-19 pandemic. Clin Med. 2020;20(3):256 261. 10.7861/clinmed.2020-0149) PMC7354033 32371418

[33] Zolk O Hafner S Schmidt CQ German Society for Experimental and Clinical Pharmacology and Toxicology (DGPT). COVID-19 pandemic and therapy with ibuprofen or renin-angiotensin system blockers: no need for interruptions or changes in ongoing chronic treatments. Naunyn Schmiedebergs Arch Pharmacol. 2020;393(7):1131 1135. 10.1007/s00210-020-01890-6) 32415494 PMC7225250

[34] BritishMedicalJournalBestPractice.Coronavirusdisease2019 (COVID-19). Available at: https://bestpractice.bmj.com/topics/en-gb/3000%20168/treatment-algorithm#referencePop126; Assessed on. 28 june, 2020.

[35] Food and Drug Administration. FDA advises patients on use of non-steroidal anti-inﬂammatory drugs (NSAIDs) for COVID-19. Available at: https://www.fda.gov/drugs/drug-safety-and-availability/fda-advises-patients-use-non-steroidal-anti-inflammatory-drugs-nsaids-covid-19; Assessed on. 28 june, 2020.

[36] European Medicines Agency. EMA gives advice on the use of non-steroidal anti-inﬂammatories for COVID-19. Available at: https://www.ema.europa.eu/en/news/ema-gives-advice-use-non-steroidal-anti-inflammatories-covid-19; Assessed on. 28 june, 2020.

[37] Bannwarth B Acetaminophen or NSAIDs for the treatment of osteoarthritis. Best Pract Res Clin Rheumatol. 2006;20(1):117 129. 10.1016/j.berh.2005.09.004) 16483911

[38] Shanthanna H Cohen SP Strand N et al. Recommendations on Chronic Pain Practice during the COVID-19 Pandemic. A Joint Statement by American Society of Regional Anesthesia and Pain Medicine (ASRA) and European Society of Regional Anesthesia and Pain Therapy (ESRA). 2020. Available at: https://www.asra.com/guidelines-articles/original-articles/covid-19-resources/covid-19-resources/legacy-b-blog-posts/2020/03/27/recommendations-on-chronic-pain-practice-during-the-covid-19-pandemic, ASRA pain medicine as guidelines on 27 March 2020. 10.1111/anae.15076 PMC7262200 32259288

[39] Sanapati J Manchikanti L Atluri S et al. Do regenerative medicine therapies provide long-term relief in chronic low back pain: a systematic review and metaanalysis. Pain Phys. 2018;21(6):515 540. 30508983

[40] Zhang J Wang JH . PRP treatment effects on degenerative tendinopathy - an in vitro model study. Muscles Ligaments Tendons J. 2014;4(1):10 17. 24932441 PMC4049643

[41] El-Tallawy SN Nalamasu R Pergolizzi JV Gharibo C . Pain management during the COVID-19 pandemic. Pain Ther. 2020;9(2):453 466. 10.1007/s40122-020-00190-4) 32840756 PMC7445106

[42] Shanthanna H Strand NH Provenzano DA et al. Caring for patients with pain during the COVID-19 pandemic: consensus recommendations from an international expert panel. Anaesthesia. 2020;75(7):935 944. 10.1111/anae.15076) 32259288 PMC7262200

[43] Centers for Medicare and Medicaid Services. CMS Releases Recommendations on Adult Elective Surgeries, Non-Essential Medical, Surgical, and Dental Procedures during COVID-19 Response. Available at: https://www.cms.gov/newsroom/press-releases/cms-releases-recommendations-adult-elective-surgeries-non-essential-medical-surgical-and-dental, USA. Last Accessed April; vol 28; 2020. 26110197

[44] Smith C King W O’Brien D Jr Laseter J Spine Intervention Society’s Patient Safety Committee’s Patient Safety Committee. Masks, gowns, and caps for interventional spine pain procedures. Pain Med. 2018;19(6):1293 1294. 10.1093/pm/pny016) 29415234

[45] Anesthesiologist ASf. UPDATE: the use of personal protective equipment by anesthesia professionals during the COVID-19 pandemic. American Society of Anesthesiologists (ASA). American Society for Anesthesiologists; 2020. Available at: https://www.asahq.org/about-asa/newsroom/news-releases/2020/03/update-the-use-of-personal-protective-equipment-by-anesthesia-professionals-during-the-COVID-19-pandemic. Accessed March 31, 2020.

